# Crystal Design and Photoactivity of TiO_2_ Nanorod Template Decorated with Nanostructured Bi_2_S_3_ Visible Light Sensitizer

**DOI:** 10.3390/ijms231912024

**Published:** 2022-10-10

**Authors:** Yuan-Chang Liang, Shao-Yu You, Bo-Yue Chen

**Affiliations:** Department of Optoelectronics and Materials Technology, National Taiwan Ocean University, Keelung 20224, Taiwan

**Keywords:** composites, vulcanization, photoactivity

## Abstract

In this study, TiO_2_-Bi_2_S_3_ composites with various morphologies were synthesized through hydrothermal vulcanization with sputtering deposited Bi_2_O_3_ sacrificial layer method on the TiO_2_ nanorod templates. The morphologies of decorated Bi_2_S_3_ nanostructures on the TiO_2_ nanorod templates are controlled by the duration of hydrothermal vulcanization treatment. The Bi_2_S_3_ crystals in lumpy filament, nanowire, and nanorod feature were decorated on the TiO_2_ nanorod template after 1, 3, and 5 h hydrothermal vulcanization, respectively. Comparatively, TiO_2_-Bi_2_S_3_ composites with Bi_2_S_3_ nanowires exhibit the best photocurrent density, the lowest interfacial resistance value and the highest photodegradation efficiency towards Rhodamine B solution. The possible Z-scheme photoinduced charge separation mechanism and suitable morphology of Bi_2_S_3_ nanowires might account for the high photoactivity of TiO_2_ nanorod-Bi_2_S_3_ nanowire composites.

## 1. Introduction

Nanorod arrays of TiO_2_ are beneficial to provide direct channels for electron transport, reducing the recombination probability of electrons in the transmission process. The rod morphology helps to improve the electron injection and collection efficiency of TiO_2_ semiconductor [1,2,3,4,5]. However, the wide band gap nature of TiO_2_ engenders poor response to visible light [6,7]. In order to improve the photoactivity of TiO_2_, the strategy of semiconductor coupling is often used to enhance the solar energy conversion and utilization of TiO_2_ [8]. Several cases of TiO_2_ coupled with a visible light sensitizer has been shown a promising approach to improve solar energy utilization efficiency. TiO_2_ microspheres coupled with CdS nanoparticles enhance the light harvesting ability and suppress the electron-hole recombination of TiO_2_ and CdS [9]. The electrospinning formed TiO_2_/CuO composite nanofibers enhance light absorbance and interparticle charge transfer and lower the band gap energy, thus promoting absorbance and utilization of photon energy from a broader light spectrum [10]. Precise control of Bi_2_O_3_ coverage layer phase composition realizes the high photoactivity of the one-dimensional TiO_2_-Bi_2_O_3_ composites [11]. The decoration of ZnFe_2_O_4_ crystallites onto TiO_2_ improves the photoactivity of TiO_2_ and enhance the photodegradation performance towards Methylene orange [12]. The TiO_2_/Bi_2_S_3_ core–shell nanowire arrays demonstrate improved photocurrent density than that of pristine TiO_2_ because of the broadened light absorption ability and the increased charge carrier separation efficiency [13]. Similarly, Bi_2_S_3_ nanowires/TiO_2_ nanorod arrays exhibit an excellent photoelectrochemical activity [14]. Rosette-rod TiO_2_/Bi_2_S_3_ shows substantial improvement in photoresponse in compared with pristine TiO_2_ photoanode [15]. Furthermore, hydrothermal method deposition of TiO_2_/Bi_2_S_3_ composite film is presented to be promising for applications of photoanode [16]. These examples clearly present the feasibility of construction of TiO_2_-Bi_2_S_3_ heterogeneous system to be used in photoactive devices with an improved efficiency.

Among various visible-light sensitizers, Bi_2_S_3_ has attracted much attention because it is environmentally friendly and non-toxic. Bi_2_S_3_ has a high optical absorption coefficient and a suitable band gap, which can absorb most of the visible light spectrum [17]. Bi_2_S_3_ crystals with a large area growth and different morphologies have been realized through various chemical routes [18]. The photoactivity of Bi_2_S_3_ crystals is highly dependent on shape, size, and crystalline quality. How to precisely control the microstructures of Bi_2_S_3_ in order to fabricate TiO_2_-Bi_2_S_3_ composites with satisfactory photoactivity is an important issue. In this study, Bi_2_S_3_ crystals with various microstructures were decorated on TiO_2_ nanorod arrays via vulcanization of Bi_2_O_3_ sacrificial layer. The vulcanization of metal oxide to obtain metal sulfide has been shown to be a promising and easy approach to synthesize the metal sulfides with controllable microstructures [17,19]. Using metal oxide sacrificial layer to form the metal sulfide through vulcanization is an easy approach to control the microstructure of the as-synthesized metal sulfide. This approach is suitable for microstructural control of the sulfide crystals and thus tuning the physical properties of the samples. However, studies on synthesis of TiO_2_-Bi_2_S_3_ composites through vulcanization of Bi_2_O_3_ layer and their photoactivities are lacking. A detailed vulcanization process-dependent microstructure evolution and photoactive properties of TiO_2_-Bi_2_S_3_ composites are proposed in this study to realize the design of TiO_2_-Bi_2_S_3_ composites with high photoactive performance.

## 2. Experiments

The synthesis of TiO_2_ nanorod template on the F-doped SnO_2_ (FTO) substrate was realized through a hydrothermal method at 170 °C. Other detailed parameters and preparation procedures have been described elsewhere [4]. The Bi_2_O_3_ thin films were sputter deposited on the TiO_2_ nanorod template using a radio-frequency magnetron sputtering system. A metallic Bi disc was used as the target (99.9 wt%, 2 inches in diameter); the substrate temperature was fixed at 410 °C. The working atmosphere is mixed Ar/O_2_ with a ratio of 1/1, the working pressure is 20 mtorr and sputtering power is 30 W. The sputtering duration is 50 min. The Bi_2_O_3_ thin-film coated TiO_2_ nanorod templates are further immersed in a 20 mL reaction solution containing 0.1M thiourea and sealed in a Teflon-lined autoclave for a hydrothermal reaction at 160 °C for 1, 3, and 5 h to obtain TiO_2_-Bi_2_S_3_ composites (named as BT-1, BT-3, and BT-5, respectively.). 

Crystallographic structures of as-synthesized samples were investigated by X-ray diffraction (XRD) analysis using Cu K*α* radiation with a two-theta scan range of 20–60° and scan rate of four degrees per min. The morphologies of the as-synthesized samples were investigated using a field emission scanning electron microscopy (FE-SEM). The morphology, high resolution images, crystallographic structure, and composition of BT-1 and BT-3 composite samples were investigated by high-resolution transmission electron microscopy (HR-TEM). X-ray photoelectron spectroscopy (XPS) was used to analyze the elemental binding energies of the as-synthesized samples. The diffuse reflectance spectra of the as-synthesized samples were recorded by using UV-vis spectrophotometer (Jasco V750) at the wavelength range of 200–800 nm. Photoelectrochemical (PEC) and electrochemical impedance spectroscopy (EIS) properties of various photoanodes were investigated using the potentiostat (SP150, BioLogic, Orlando, FL, USA). During measurements, an Ag/AgCl electrode were used as the reference electrode herein. The 0.5 M Na_2_SO_4_ solution was used as electrolyte and the light irradiation source for measurements is excited from 100 W Xe arc lamp. The 10 mL RhB solution (5 × 10^−5^ M) was used as target dye solution for photodegradation experiments. The RhB solution containing various photocatalysts with different irradiation durations (0, 15, 30, 45, and 60 min) was carried out to understand the photocatalytic activity of various as-synthesized samples.

## 3. Results and Discussion

**Figure 1:**
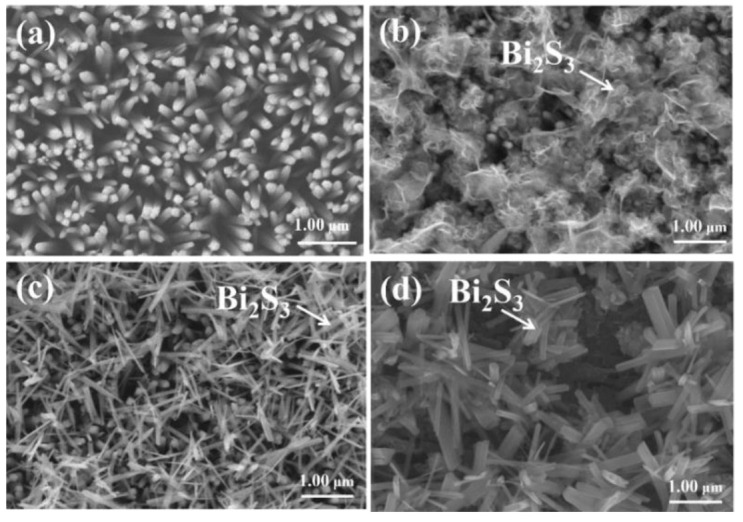
SEM images: (**a**) TiO_2_ template, (**b**) BT-1, (**c**) BT-3, and (**d**) BT-5.

The morphology of initial pristine TiO_2_ rod array template was clearly observed from the SEM images in Figure 1a. As seen, the TiO_2_ rods are uniformly grown on the entire substrate; moreover, TiO_2_ rods have a regular cross-section and the side-wall surfaces are smooth. Figure 1b displays the morphology of BT-1. A large number of lumpy filament-like crystals are covered on the top region of the TiO_2_ rod template. Figure 1c shows that abundant nanowires with around 0.8 μm length were grafted on the TiO_2_ rod template for the BT-3 sample. Such a morphology is similar to that of Bi_2_S_3_ nanowire-decorated TiO_2_ rod heterostructures synthesized via a facile two-step hydrothermal growth process reported by Liu et al. [14]. Compared to Figure 1c, after extending the hydrothermal duration to 5 h, the diameter of one dimensional Bi_2_S_3_ thickened, and the length increases from 0.8 μm to 1.2 μm as exhibited in Figure 1d. Some aggregations of one-dimensional Bi_2_O_3_ crystals appear on the TiO_2_ rod template. It has been shown that Bi_2_O_3_ oxide layer is easily etched and transfer to Bi_2_S_3_ phase during a vulcanization process with sulfur ions in the reaction solution [20].

**Figure 2:**
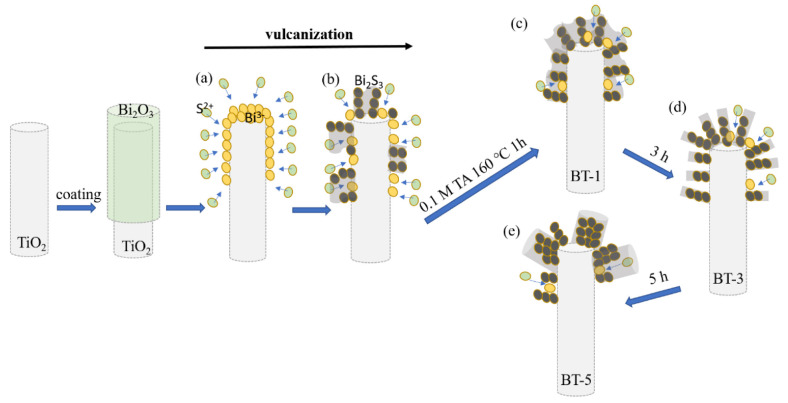
Schematic illustration of the formation processes of Bi_2_S_3_ nanostructures.

Figure 2 shows the possible formation processes of Bi_2_S_3_ nanostructures on the TiO_2_-Bi_2_O_3_ composite rod template via different vulcanization processes in this study. Notably, in aqueous solution the solubility of Bi_2_S_3_ is much lower than that of Bi_2_O_3_. When the TiO_2_-Bi_2_O_3_ composite rods are placed in a reaction solution containing a large amount of S^2−^ ions, there is a high possibility of S^2−^ ions transfer toward to the Bi_2_O_3_ surface as exhibited in (a). Furthermore, due to solubility disparity, the formation of Bi_2_S_3_ nuclei on Bi_2_O_3_ will occur during the vulcanization process because of the ion exchange of S^2−^ from the sulfur precursor solution and O^2−^ from the Bi_2_O_3_ surface. The generation of Bi_2_S_3_ crystals will continually proceed with a result of Bi^3+^ reacting with S^2−^ as exhibited in schematic (b). Further extending reaction duration, the Bi_2_S_3_ nuclei will slowly stack up to form loose filament structure at the given vulcanization condition of schematic (c). When an increase of the vulcanization reaction duration to 3 h, the loose and lumpy Bi_2_S_3_ clusters will transfer into abundant, distinguishable, and separable nanowire crystals covered on the TiO_2_ template as displayed in schematic (d). When the reaction duration reaches 5 h, the one-dimensional Bi_2_S_3_ crystals continue to aggregate into a thicker rod structure, as shown in schematic (e).

**Figure 3:**
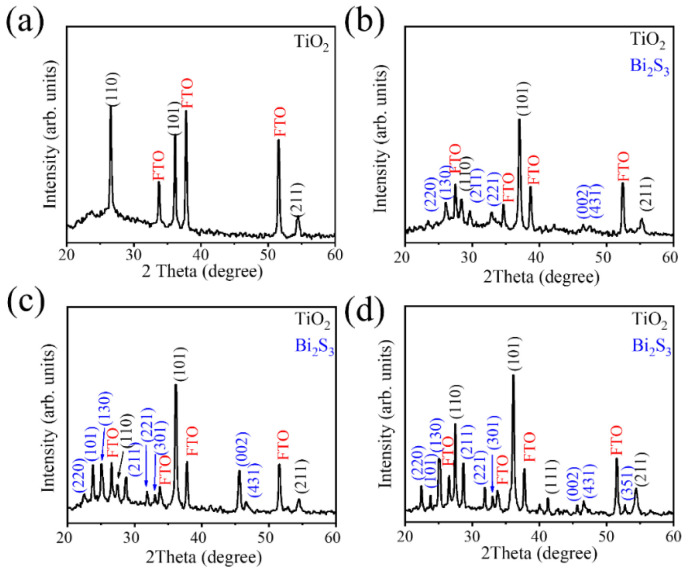
XRD patterns: (**a**) TiO_2_ template, (**b**) BT-1, (**c**) BT-3, and (**d**) BT-5.

Figure 3a–c demonstrate the XRD patterns of vulcanization-treated BT-1, BT-3, and BT-5 samples. In addition to the Bragg reflections originated from the FTO substrate, the characteristic diffraction peaks centered at approximately 26.61°, 36.18°, and 54.76° can be indexed to (110), (101), and (211) planes of rutile TiO_2_ (JCPDS No. 00-021-1276), revealing high crystallinity of the TiO_2_ template. Notably, after hydrothermal vulcanization, several visible Bragg reflections originated from orthorhombic Bi_2_S_3_ phase could be clearly observed (JCPDS No. 017-0320) in the BT-1, BT-3, and BT-5 samples. Figure 3a–c present that addition of thiourea in hydrothermal reaction solution can vulcanize the Bi_2_O_3_ sacrificial oxide layer to transfer into the corresponding sulfide phase in BT-1, BT-3, and BT-5. The prolonged vulcanization process (from 1 to 5 h) improves Bragg reflection intensity of Bi_2_S_3_ phase of the samples, revealing an improved crystallinity of the samples. This might be associated with crystal morphology transformation of the loose and lumpy filament-like Bi_2_S_3_ crystals of BT-1 to one-dimensional Bi_2_S_3_ rods of BT-5 with prolonged vulcanization process. No Bragg reflections associated with Bi_2_O_3_ and other impurity phases were detected in the XRD patterns, revealing the vulcanization processes herein successfully synthesized the TiO_2_-Bi_2_S_3_ composite structure, and the as-synthesized composite structures are crystalline.

**Figure 4:**
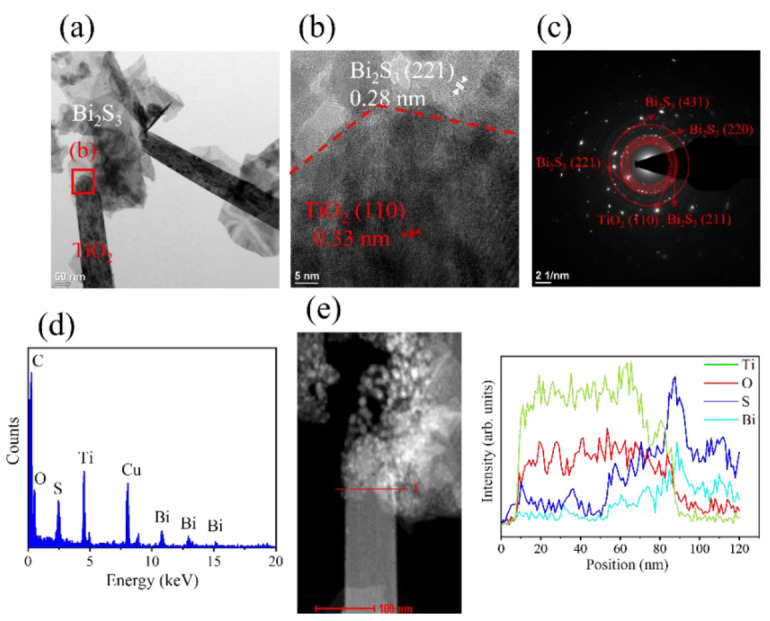
(**a**) Low magnification TEM image of BT-1. (**b**) HRTEM image taken from the local region of the sample in (**a**). (**c**) The SAED pattern of BT-1 in (**a**). (**d**) EDS spectrum taken from the sample in (**a**). (**e**) EDS line-scanning profiles across the composite.

Figure 4a shows the low-magnification TEM image of the BT-1. It can be observed that Bi_2_S_3_ flakes decorated on the TiO_2_ rod. Figure 4b is the HRTEM image taken from the red square in Figure 3a. The distinct lattice fringes with a distance of approximately 0.28 nm are from the interplane spacing of orthorhombic Bi_2_S_3_ (2 2 1) and the lattice fringes with a spacing of 0.33 nm are from the interplane spacing of tetragonal TiO_2_ (1 1 0). Figure 4c showed the selected area electron diffraction (SAED) pattern of several BT-1. Several clear diffraction spots arranged in centric rings are associated with the orthorhombic Bi_2_S_3_ (2 1 1), (2 2 1), and (4 3 1) planes and the rutile TiO_2_ (1 1 0) plane, which agrees with the result observed by XRD analysis. This demonstrates the well-constructed TiO_2_-Bi_2_S_3_ heterogeneous structure of BT-1, and a good crystallinity of the composites. The EDS spectrum in Figure 4d shows Ti, O, Bi, and S are the main elements in this composite structure. Furthermore, the particular element line scan intensity distributions displayed in Figure 4e reveals the construction of Bi_2_S_3_ flakes on the TiO_2_ rod for the BT-1.

**Figure 5:**
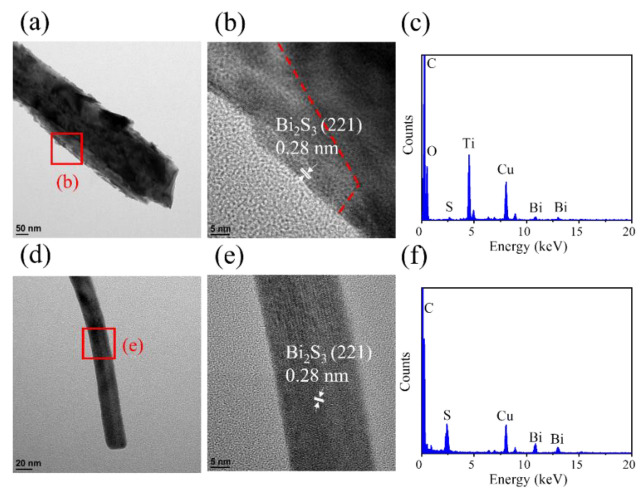
(**a**) Low-magnification TEM image of scratched BT-3. (**b**) HRTEM image taken from the local region of the sample in (**a**). (**c**) The EDS spectrum of BT-3 in (**a**). (**d**) Low magnification TEM image of Bi_2_S_3_ nanowire scratched from BT-3. (**e**) HRTEM image taken from the local regions of the sample in (**d**). (**f**) The EDS spectrum of Bi_2_S_3_ nanowire in (**d**).

Figure 5a shows the low-magnification TEM image of the BT-3 structure scratched from the sample. The sidewall of the composite showed an undulated feature because of the decoration of Bi_2_S_3_ crystals. However, a broken top region of the structure was observed in Figure 5a. Because the preparation of TEM sample with a scratched off method will destroy the integrity of the BT-3 composite, the distinct two-layered structure of the BT-3 composite is not observed herein, as revealed in the aforementioned SEM observation. Figure 5b displays the HRTEM image taken from the red square region in Figure 5a. The distinct lattice fringes with a distance of approximately 0.28 nm are associated with the interplanar spacing of Bi_2_S_3_ (2 2 1). Figure 5c demonstrates the EDS spectrum taken from the nanostructure in Figure 5a. In addition to Cu and C originated from the TEM grid, the Ti, O, Bi, and S are the main constituent elements in this composite structure, which proves that TiO_2_ and Bi_2_S_3_ phases coexist in the composite structure. Figure 5d presents a low-magnification image of the scratched nanowire structure from BT-3 sample. The nanowire has the diameter of 23 nm, and the surface is smooth. The nanowire shows a little bent state. Figure 5e shows the HRTEM image taken from the red square in Figure 5d. The distinct and ordered lattice fringes shows the single crystalline quality of the Bi_2_S_3_ nanowire. Similarity, a distinguishable Bi_2_S_3_ (2 2 1) lattice image in the one-dimensional Bi_2_S_3_ crystal in an orthorhombic structure has been shown in WO_3_/Bi_2_S_3_ composite synthesized via chemical bath deposition [21]. Figure 5f shows the EDS spectrum taken from the single Bi_2_S_3_ nanowire, where the EDS spectrum indicates high purity of Bi_2_S_3_ composition of the nanowire.

**Figure 6:**
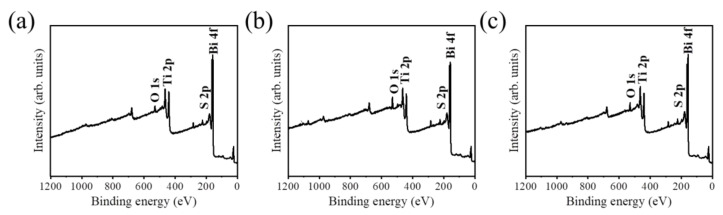
XPS survey scan spectra: (**a**) BT-1, (**b**) BT-3, and (**c**) BT-5.

**Figure 7:**
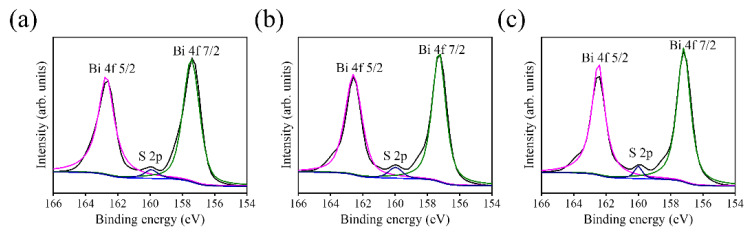
XPS analysis of narrow scan Bi 4f spectra: (**a**) BT-1, (**b**) BT-3, and (**c**) BT-5.

Figure 6 shows the characteristic XPS spectral lines of various samples. The main constituent elements of Bi, S, Ti, and O are detected in BT-1, BT-3, and BT-5, supporting the existence of Bi_2_S_3_ and TiO_2_ in the composite structure. Moreover, the relatively weak Ti signals from the spectra in comparison with that of the Bi signals, revealing the capping layer of Bi_2_S_3_ phase on the TiO_2_ template for the test samples, and this spectral feature has widely been observed in the composite structure, having obvious layering characteristics [9]. In addition to the C signal that originated from the sample contamination on exposure to ambient air, no impurity was detected in the as-synthesized samples. In order to further investigate the elemental binding states of the Bi_2_S_3_ capping layer, the XPS narrow scan spectra of Bi 4f for BT-1, BT-3, and BT-5 are displayed in Figure 7a–c, respectively. Two sharp and distinct peaks centered at approximately 157.3 eV and 162.6 eV, which are assigned to Bi 4f_7/2_ and Bi 4f_5/2_ of Bi_2_S_3_, respectively [22]. The tiny peak located between the two distinct characteristic peaks of Bi 4f is originated from S 2p. The binding energies of Bi 4f core-level peaks corresponded to the characteristic binding state of Bi^3+^ in the Bi_2_S_3_, revealing the well formation of the Bi_2_S_3_ phase through vulcanizing the Bi_2_O_3_ layer. No metallic Bi or Bi_2_O_3_ appeared after vulcanization.

**Figure 8:**
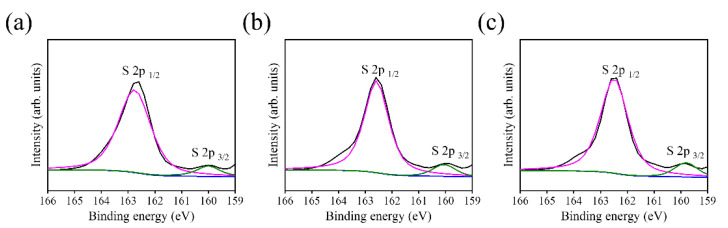
XPS analysis of narrow scan S 2p spectra: (**a**) BT-1, (**b**) BT-3, and (**c**) BT-5.

Notably, the narrow scan spectra of S 2p region of BT-1, BT-3, and BT-5 in Figure 8 reveal the characteristic peaks centered at approximately 162.6 eV and 160.1 eV, which are assigned to S 2p_1/2_ and S 2p_3/2_, respectively. The S 2p binding energies herein associated with the aforementioned Bi 4f binding states evidenced the formation of Bi-S bonds in the capping layer synthesized via the given vulcanization processes in this study [23].

**Figure 9:**
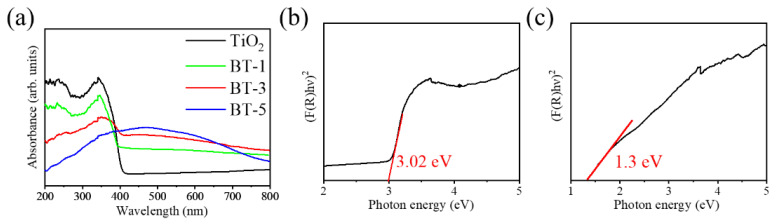
(**a**) UV–vis absorbance spectra of various samples. Band gap evaluation of (**b**) pristine TiO_2_ and (**c**) pristine Bi_2_S_3_.

A comparison of the UV-vis absorption spectra of TiO_2_ template and various TiO_2_-Bi_2_S_3_ samples are displayed in Figure 9a. The absorption of the TiO_2_ template is mainly in the UV light range. The decoration of Bi_2_S_3_ extends the absorption range of the TiO_2_ template to the visible light range because of the narrow energy gap of Bi_2_S_3_. This is consistent with the feature of optical absorption spectra for Bi_2_S_3_ nanorod/TiO_2_ nanoplate composites [24]. Moreover, in the previous TiO_2_/Bi_2_S_3_ core–shell nanowire arrays synthesized via a successive ionic layer adsorption and reaction method, the absorption of the composite extends to cover the visible light range, and the decoration of Bi_2_S_3_ phase increases the light absorption ability of the pristine TiO_2_ nanowires [8]. Notably, the band gap energies of the pristine TiO_2_ and Bi_2_S_3_ are evaluated in Figure 9b,c, respectively, from Tauc plots based on Kubelka–Munk function [25]. The TiO_2_ has a band gap energy of approximately 3.02 eV, and the band gap energy of the reference Bi_2_S_3_ derived from the sputtering deposited Bi_2_O_3_ layer and then vulcanized using 0.1 M thiourea is approximately 1.3 eV. Based on the evaluated band gap energy, the construction of the TiO_2_/Bi_2_S_3_ heterostructure herein improves the light harvesting ability.

**Figure 10:**
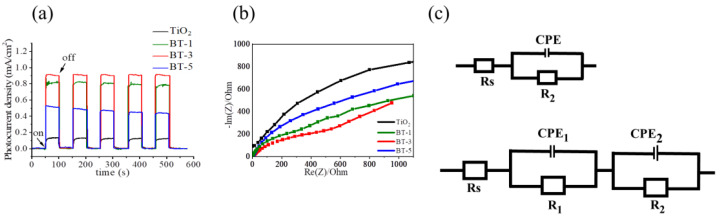
(**a**) Photocurrent density versus time curves of various samples at 0.5 V vs. Ag/AgCl under chopped illumination. (**b**) Nyquist plot of various samples at an open-circuit potential under illumination (**c**) Possible equivalent circuits used to fit R2 value of the pristine TiO_2_ and TiO_2_-Bi_2_S_3_ composite.

Figure 10a shows the transient photocurrent performance of various photoelectrodes under repeated on/off irradiation cycles at 0.5 V (vs. Ag/AgCl). It is known that the transient photocurrent performance is highly associated with the photoresponse, charge carrier transport speed, charge carrier separation efficiency, and charge carrier recombination rate of samples. When the light is on, the photocurrent densities of all samples swiftly rise to a stable value of 0.12, 0.81, 0.93, and 0.47 mA/cm^2^ for TiO_2_, BT-1, BT-3, and BT-5, respectively. When the light is off, the photocurrent densities of the samples drop to their initial dark current density value instantaneously. Such a fast rise and fall process of the photocurrents indicates that carrier transport and separation in the prepared photoelectrodes proceed quickly [11]. Furthermore, the photoresponse of the BT-3 increased approximately three times higher than that of the TiO_2_ template. The BT-1 and BT-5 also exhibited enhanced photoresponses in comparison with the TiO_2_ template. Among various composite structure, the BT-3 has the highest photocurrent response, indicating it has the lowest photogenerated electron/hole recombination rate. By contrast, the BT-5 exhibits a lower photocurrent response among various composites. This might be attributed to the following reason. Substantially excessive Bi_2_S_3_ deposition leads to an increase in the number of recombination centers, and as a result, a decrease in photocurrent density has been observed due to the loss of photogenerated electrons [15]. The results herein prove that decoration of Bi_2_S_3_ crystals onto the TiO_2_ template can effectively improve the photoinduced charge separation efficiency of the TiO_2_ template, resulting in more efficient charge migration and higher photocurrent density of the TiO_2_-Bi_2_S_3_ composite structure. A similar improved transient photocurrent performance because of decoration of visible light sensitizer onto the wide band gap semiconductor with a suitable band alignment has been demonstrated in TiO_2_-Bi_2_O_3_ and ZnO-Sn_2_S_3_ heterostructures [11,25]. Figure 10b presents the Nyquist plots of various photoelectrodes. The semicircular radius of the Nyquist plots can reflect the charge transfer resistance of the photoelectrode materials. As show in Figure 10b, the arc radius of the TiO_2_-Bi_2_S_3_ composites herein are smaller than that of the TiO_2_ template, revealing that formation of TiO_2_/Bi_2_S_3_ heterojunction is beneficial for interfacial charge transfer. Comparatively, BT-3 has the smallest radius of the Nyquist curve among various samples. The possible equivalent circuits shown in Figure 10c are used to fit the EIS Nyquist results, where Rs, R1, R2, and CPE represent the solution resistance, semiconductor depletion layer resistance, charge transfer resistance, and chemical capacitance, respectively [26]. Herein, the R2 is evaluated from the fitting results of the Nyquist plots at the low frequency region. The R2 values of the pristine TiO_2_, BT-1, BT-3, and BT-5 are 3500, 583.3, 463.4, and 925 Ω, respectively. The BT-3 exhibited the smallest R2 value. As the vulcanization duration was further prolonged, a larger size of Bi_2_S_3_ crystallites deposition occurred, which might prolong the electron diffusion path in the BT-5 composite, resulting a larger R2 value among various composites. Notably, charge transfer resistances of the BT-1, BT-3, and BT-5 are much lower than that of TiO_2_. This indicates that decoration of Bi_2_S_3_ onto the TiO_2_ could effectively reduce the photoinduced charge transfer resistance of the TiO_2_ template and enhance the separation efficiency of charge carriers. Both the transient photocurrent and Nyquist plot results herein support an effective separation of photogenerated electron–hole pairs and faster interfacial charge transfer occurred on the BT-3 interface, which might lead to the enhanced photocatalytic performance.

**Figure 11:**
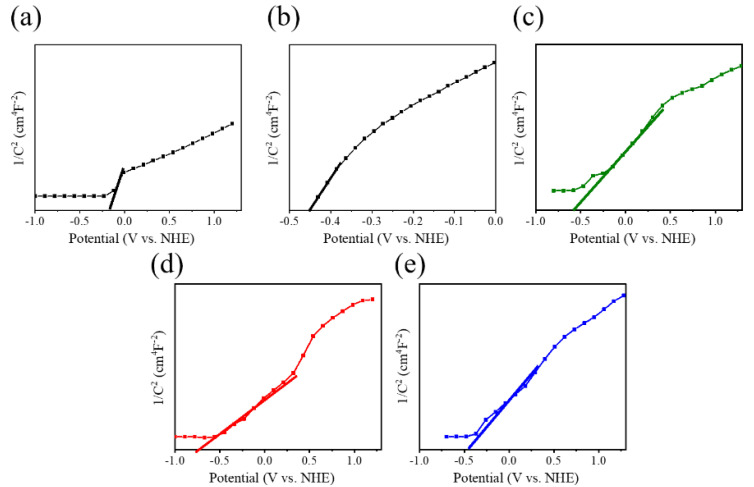
Mott–Schottky plots of various samples: (**a**) pristine TiO_2_, (**b**) pristine Bi_2_S_3_, (**c**) BT-1, (**d**) BT-3, and (**e**) BT-5.

Figure 11 shows the Mott–Schottky (M–S) plots of TiO_2_, Bi_2_S_3_, and TiO_2_-Bi_2_S_3_ composites performed at 1 kHz. All the samples were measured with a positive slope in the M–S plots, revealing an n-type nature of the composed semiconductors. The flat band potential (E_fb_) of various samples can be calculated from the x intercept of the linear region in the M–S plots according to the M–S equation [27]. Furthermore, the normal hydrogen electrode (NHE) potential can be converted from the Ag/AgCl reference electrode as NHE = V(Ag/AgCl)—0.197 V. The E_fb_ of TiO_2_ and Bi_2_S_3_ referenced samples are −0.21 and −0.45 V vs. NHE, respectively. Comparatively, the flat band potential of the TiO_2_-Bi_2_S_3_ composites has a negative shift compared to the TiO_2_ template as exhibited in Figure 11c–e. Moreover, the tangent slope in the linear region of the M–S plots for the TiO_2_-Bi_2_S_3_ composites is smaller than the TiO_2_ template, demonstrating the decoration of Bi_2_S_3_ crystallites onto the TiO_2_ template increases carrier concentration and reduce the charge recombination rate of the composites. A more negative shift of the flat band potential with respective to TiO_2_ template is observed for the BT-3 among various TiO_2_-Bi_2_S_3_ composites; moreover, the smaller tangent slop in the linear region of the M–S plot was observed for the BT-3. These reveal a superior electronic property of the BT-3 than that of other TiO_2_-Bi_2_S_3_ composites. It has been shown that the crystallite size and number of visible-light sensitizers affect the photoinduced charge separation efficiency of the visible-light sensitizer-decorated TiO_2_ [28]. Moreover, the crystal quality of the semiconductor has been proved to affect its photoactivity [29]. The BT-3 because of suitable Bi_2_S_3_ crystallite quality and size shows the superior photogenerated carrier density and effective interface transfer ability among various composites in this study.

**Figure 12:**
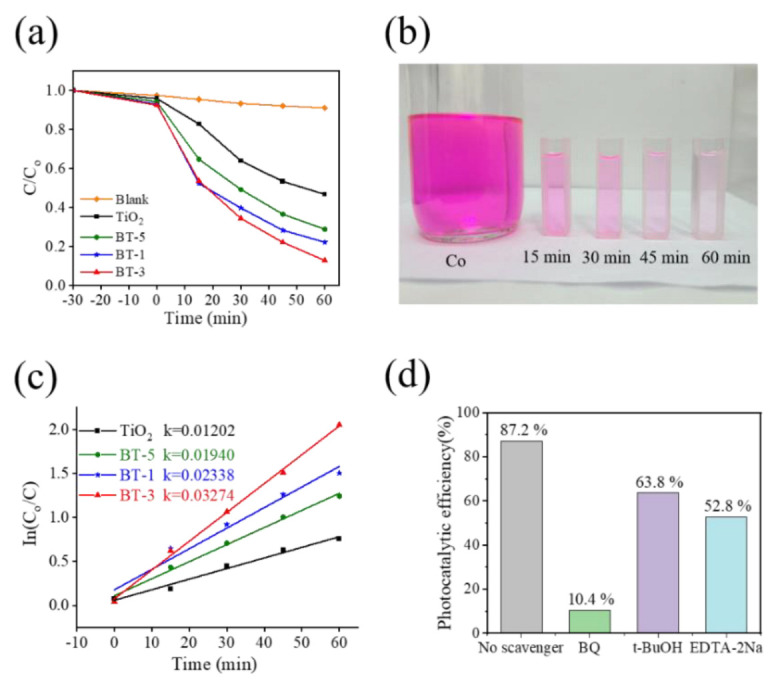
(**a**) C/Co versus irradiation duration plot. (**b**) Discoloration of RhB solution with BT-3 under different irradiation durations. (**c**) ln (Co/C) versus irradiation duration plot. (**d**) The photocatalytic performance after adding various scavengers in BT-3/RhB solution.

Figure 12a shows the photodegradation level of various photocatalysts towards RhB solution. The percentage of photodegradation was calculated using the C/Co = It/Io. The Co and C are the initial and residual concentration of the RhB solution at t = 0 and at irradiation duration t, respectively, and can be evaluated from the intensity variation of absorbance spectra of the RhB solution with and without photocatalytic reaction [30]. Figure 12a demonstrates that the BT-1, BT-3, BT-5, and TiO_2_ degrades 60.3%, 65.6%, 50.8%, and 36.1% of RhB solution, respectively, after 30 min irradiation. Furthermore, the degradation level of RhB solution reached 77.8%, 87.2%, 71.2%, and 57.1% after 60 min irradiation for the BT-1, BT-3, BT-5, and TiO_2_, respectively. Notably, the dark balanced absorptions of various samples are also conducted to understand the initial catalysts’ surface dye absorption efficiency. The C/Co of the BT-1, BT-3, BT-5, and TiO_2_ after 30 min dark balanced adsorption is 7.1%, 7.6%, 5.8%, and 4.2%, respectively. Comparatively, the BT-1 and BT-3 exhibit a slightly larger surface dye adsorption ability than that of BT-5 and TiO_2_. Figure 12b demonstrates the discoloration of RhB solution with BT-3 under different irradiation durations. Apparent discoloration appeared in the RhB solution containing BT-3 with an increased irradiation duration. To quantitatively compare the photocatalytic activity of the as-prepared photocatalyst samples, the photodegradation data were fitted to the pseudo-first-order kinetics equation: ln(Co/C) = kt, where k is the apparent first-order rate constant [4]. Figure 12c presents the plot of ln(Co/C) versus t for various samples. The higher k value is observed for the TiO_2_-Bi_2_S_3_ composites than that of pristine TiO_2_. Moreover, the BT-3 demonstrates the highest k value of 0.03274 min^−1^ among various samples, confirming the higher photocatalytic activity of BT-3 composite. The suitable morphology and decoration content of Bi_2_S_3_ crystallites on the TiO_2_ template might account for the observed result. This has also been supported by visible-light CuO sensitizer decorated ZnO composite photocatalysts that crystal morphology and content of the CuO substantially affect the photocatalytic activity of CuO-ZnO nanocomposites [31]. Active groups of the photocatalytic degradation reaction were explored by the addition of free radical capture agents, as exhibited in Figure 12d. Herein, benzoquinone (BQ, ·O_2_^−^ radical scavenger), tertiary butyl alcohol (t-BuOH, ·OH radical scavenger), and EDTA-2Na (EDTA-2Na, h^+^ radical scavenger) are used to explore the active groups of the photocatalytic degradation reaction. Notably, the photocatalytic efficiency of RhB solution with BT-3 was significantly inhibited after adding BQ, indicating that ·O_2_^−^ is the main active substance for RhB degradation. Furthermore, the results herein reveal that a single scavenger could not completely prevent the dye degradation, and the h^+^ and ·OH also contribute some degrees of photodegradation towards RhB dyes.

**Figure 13:**
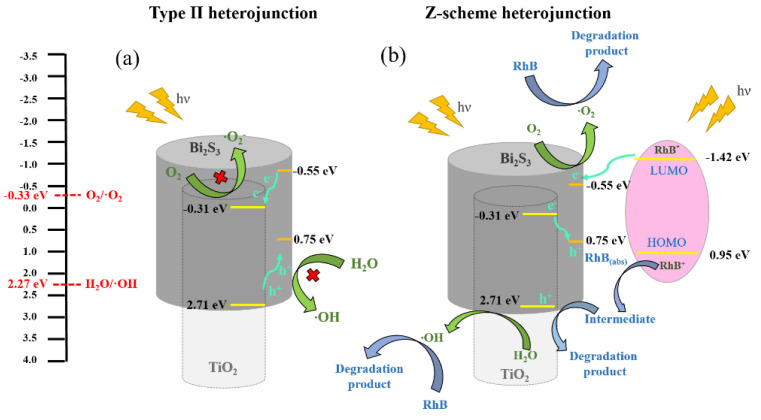
Schematic diagrams of the photogenerated electron–hole separation process for TiO_2_/Bi_2_S_3_ composites: (**a**) Type II heterojunction and (**b**) Z-scheme heterojunction.

In the M-S analysis, the flat band potentials of the TiO_2_ and Bi_2_S_3_ are −0.21 and −0.45 eV, respectively. Furthermore, the E_CB_ bottoms of the TiO_2_ and Bi_2_S_3_ can be evaluated to be −0.31 and −0.55 eV, respectively [32]. The valence band (VB) positions of the TiO_2_ and Bi_2_S_3_ are evaluated to be 2.71 and 0.75 eV, respectively. According to the band structures of TiO_2_ and Bi_2_S_3_, there are two possible migration mechanisms of photoinduced charge carriers in the TiO_2_/Bi_2_S_3_ heterojunction as shown in Figure 13. However, the potential of O_2_/·O_2_^−^ is −0.33 eV, which is more negative than CB of TiO_2_, and CB electrons are not easy to reduce O_2_. At the same time, the photoinduced holes on Bi_2_S_3_ VB cannot oxidize H_2_O to produce ·OH radicals, because the VB edge potential of Bi_2_S_3_ is more negative than the potential of H_2_O/OH (2.27 V), as shown in Figure 13a. Based on the aforementioned discussions, type II photodegradation mechanism is not suitable for the as-synthesized TiO_2_-Bi_2_S_3_ composites in this study. By contrast, a direct Z-scheme mechanism over the TiO_2_/Bi_2_S_3_ heterostructure can be proposed in Figure 13b. Upon irradiation, TiO_2_/Bi_2_S_3_ absorbs light greater than its band gap, electron-hole pairs are generated (Equations (1) and (2)), and RhB will also be excited to RhB* by light irradiation at the same time (Equation (3)). The RhB* injects electrons into the CB of Bi_2_S_3_ (Equation (4)). A similar phenomenon of electron injection from RhB* to the TiO_2_ CB has been proposed in the literature [33]. The electrons in the TiO_2_ CB position could migrate to the Bi_2_S_3_ VB, resulting in the effective separation of photoinduced charge carriers (Equation (5)). The RhB^+^ reacts with adsorbed dye to form intermediate products by the photosensitization process (Equation (6)). Parts of the holes in the TiO_2_ VB will react with the intermediate product to degrade RhB dyes (Equation (7)). Moreover, the residual photoinduced holes in the TiO_2_ VB will react with the H_2_O molecules to generate the ·OH radicals (Equation (8)), and the electrons are gathered on Bi_2_S_3_ CB to produce ·O_2_^−^ radicals (Equation (9)) [34]. Therefore, the RhB dyes are effectively photodegraded with BT-3 under irradiation (Equation (10)). A similar Z-scheme mechanism is shown in other TiO_2_-based composite systems decorated with visible-light sensitizers [35,36,37].

(1) $${\mathrm{TiO}}_{2}+h\upsilon \to {\mathrm{TiO}}_{2}{\;e}^{-}\left(\mathrm{CB}\right)+{\mathrm{TiO}}_{2}{\;h}^{+}\left(\mathrm{VB}\right)$$

(2) $${\mathrm{Bi}}_{2}S_{3}+h\upsilon \to {\mathrm{Bi}}_{2}S_{3}{\;e}^{-}\left(\mathrm{CB}\right)+{\mathrm{Bi}}_{2}S_{3}{\;h}^{+}\left(\mathrm{VB}\right)$$

(3) $$\mathrm{RhB}+h\upsilon \to {\;\mathrm{RhB}}^{*}\left(\mathrm{LUMO}\right)+{\;\mathrm{RhB}}^{+}\left(\mathrm{HOMO}\right)$$

(4) $${\mathrm{RhB}}^{*}\left(\mathrm{LUMO}\right)+{\mathrm{Bi}}_{2}S_{3}{\;e}^{-}\left(\mathrm{CB}\right)\to {\mathrm{Bi}}_{2}S_{3}{\;\mathrm{total}\;e}^{-}\left(\mathrm{CB}\right)$$

(5) $${\mathrm{TiO}}_{2}{\;e}^{-}\left(\mathrm{CB}\right)+{\mathrm{Bi}}_{2}S_{3}{\;h}^{+}\left(\mathrm{VB}\right)\to \mathrm{recombination}$$

(6) $${\mathrm{RhB}}^{+}\left(\mathrm{HOMO}\right)+{\mathrm{RhB}}_{\left(\mathrm{abs}\right)}\to \mathrm{Intermediate}$$

(7) $$\mathrm{Intermediate}+{\mathrm{TiO}}_{2}{\;h}^{+}\left(\mathrm{VB}\right)\to \mathrm{degradation}\;\mathrm{products}$$

(8) $${\mathrm{TiO}}_{2}{\;h}^{+}\left(\mathrm{VB}\right)+H_{2}O\to \cdot \mathrm{OH}$$

(9) $${\mathrm{Bi}}_{2}S_{3}{\;e}^{-}\left(\mathrm{CB}\right)+O_{2}\to \cdot O_{2}{}^{-}$$

(10) $$\mathrm{RhB}+\cdot O_{2}{}^{-}\to \mathrm{degradation}\;\mathrm{products}$$

## 4. Conclusions

TiO_2_ nanorod array coated with Bi_2_O_3_ layer was used to vulcanize to form TiO_2_-Bi_2_S_3_ composites with various morphologies. The hydrothermal vulcanization duration profoundly affects the microstructure, optical properties, and photoactivity of TiO_2_-Bi_2_S_3_ composites. The decorated Bi_2_S_3_ crystals changed the morphology from filament, nanowire to nanorod with an increased vulcanization duration from 1, 3, and 5 h, respectively. The decoration of Bi_2_S_3_ crystals enhanced the light absorption capacity of the TiO_2_ nanorod template. The improved photodegradation performance of the composites can be reasonably attributed to the construction of the direct Z-scheme heterojunction between the TiO_2_ and Bi_2_S_3_. This study demonstrates that design of TiO_2_-Bi_2_S_3_ composites with suitable morphology of decorated Bi_2_S_3_ crystals can tune the photoactivity of the composites, and the findings in this study may be of great value for the development of oxide-sulfide composites for ideal photosensitive device applications.

## Data Availability

Not applicable.
